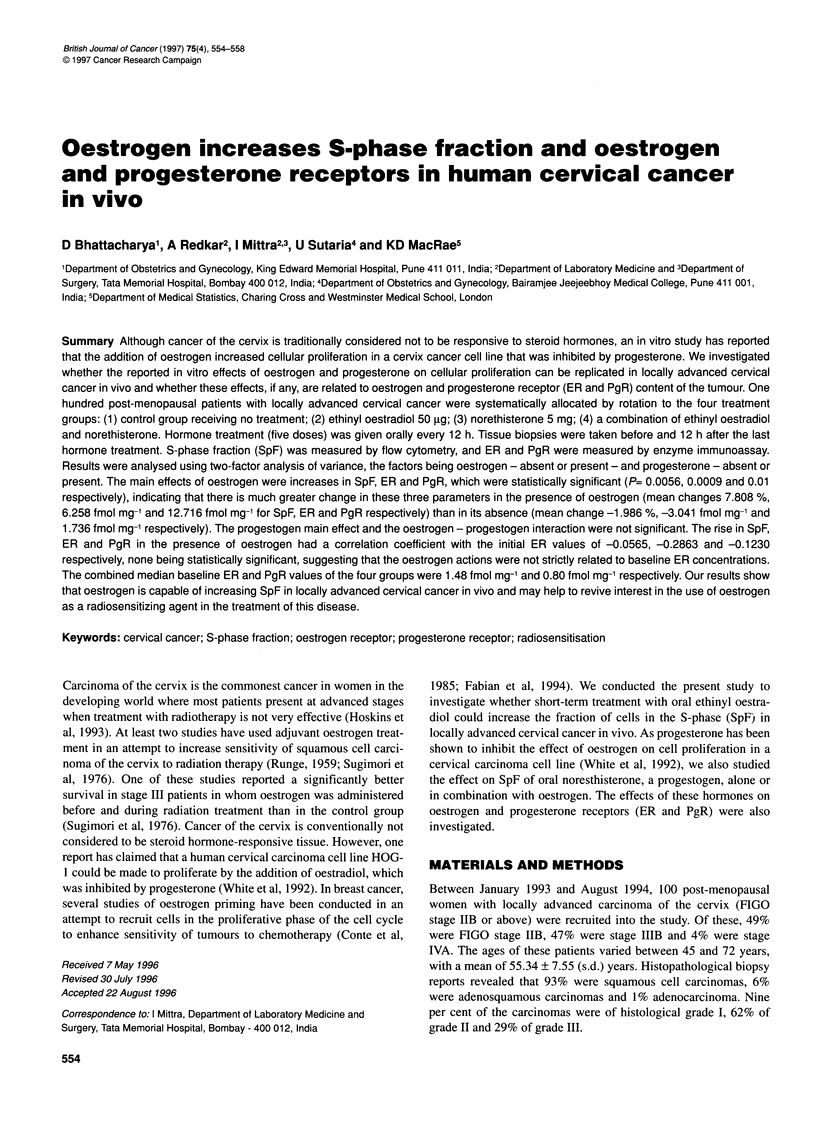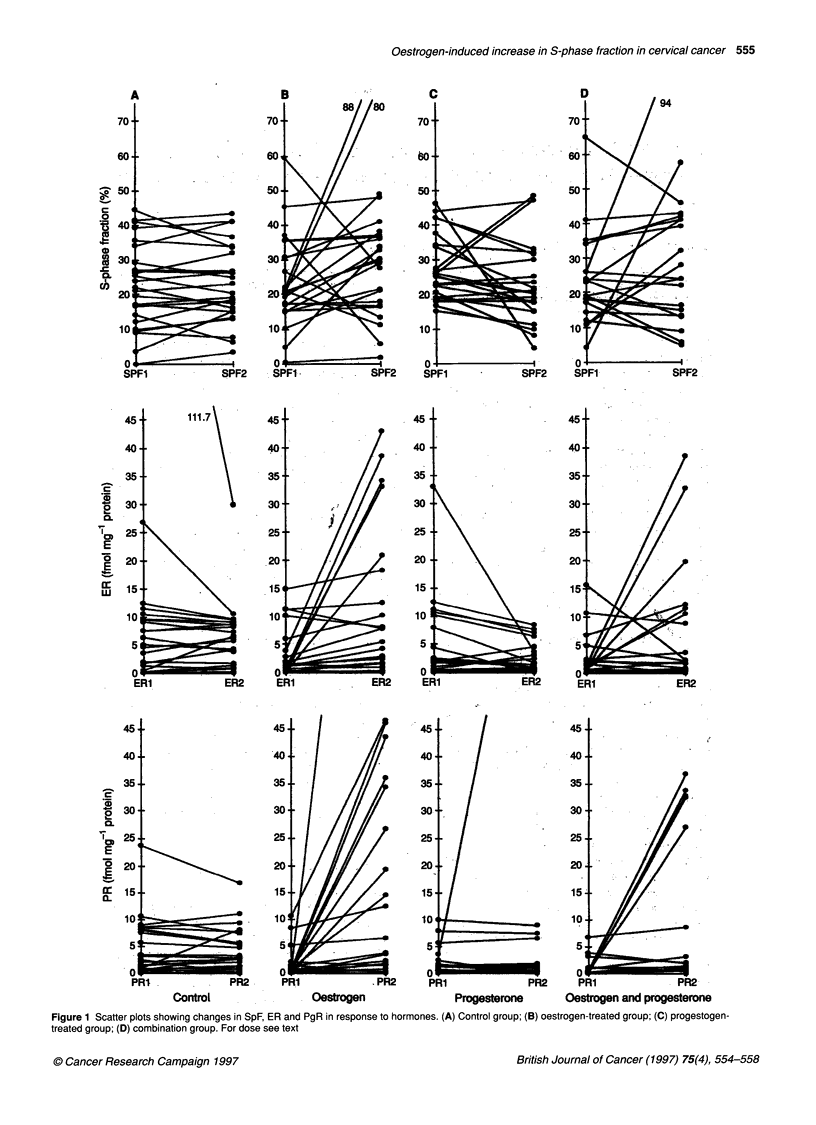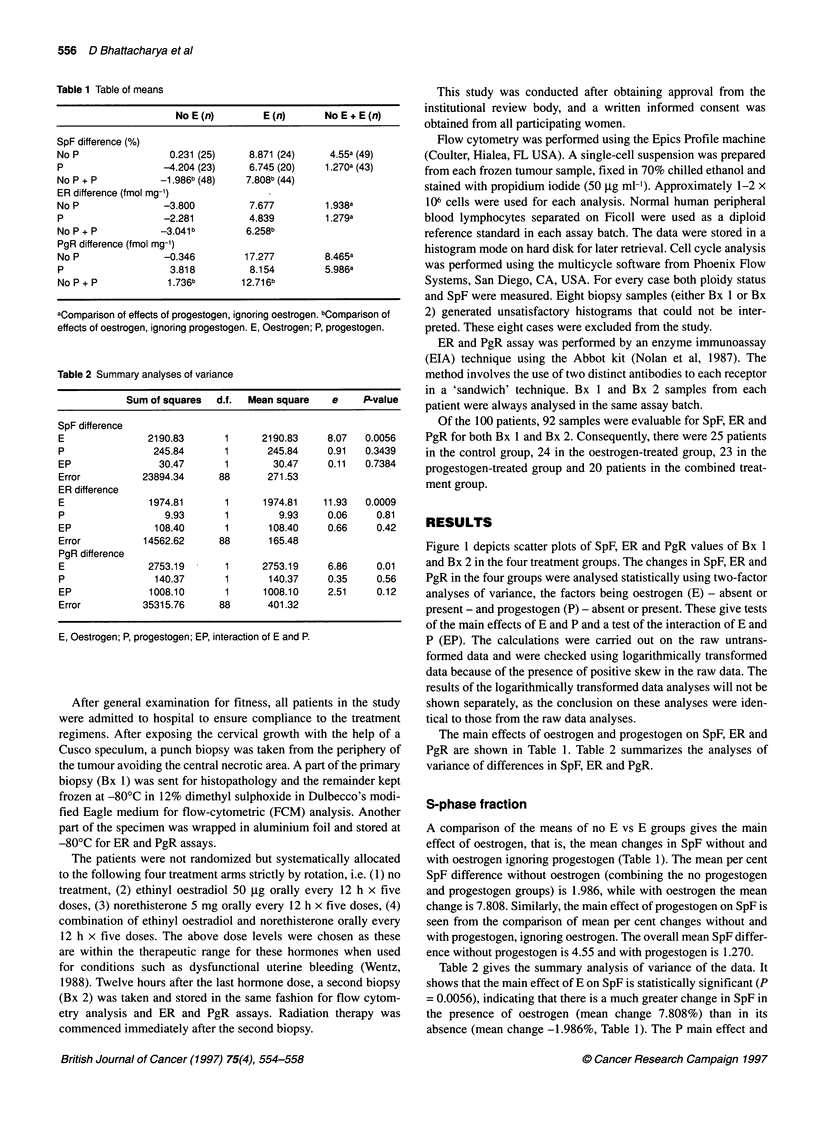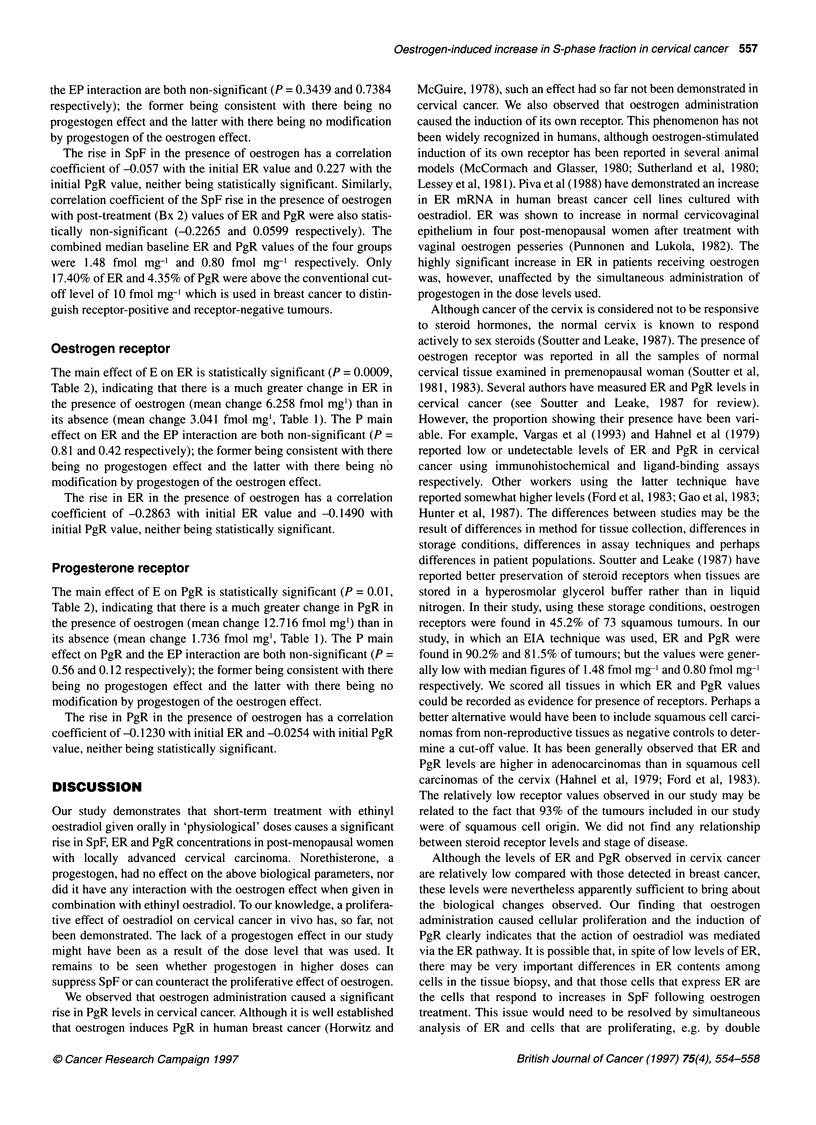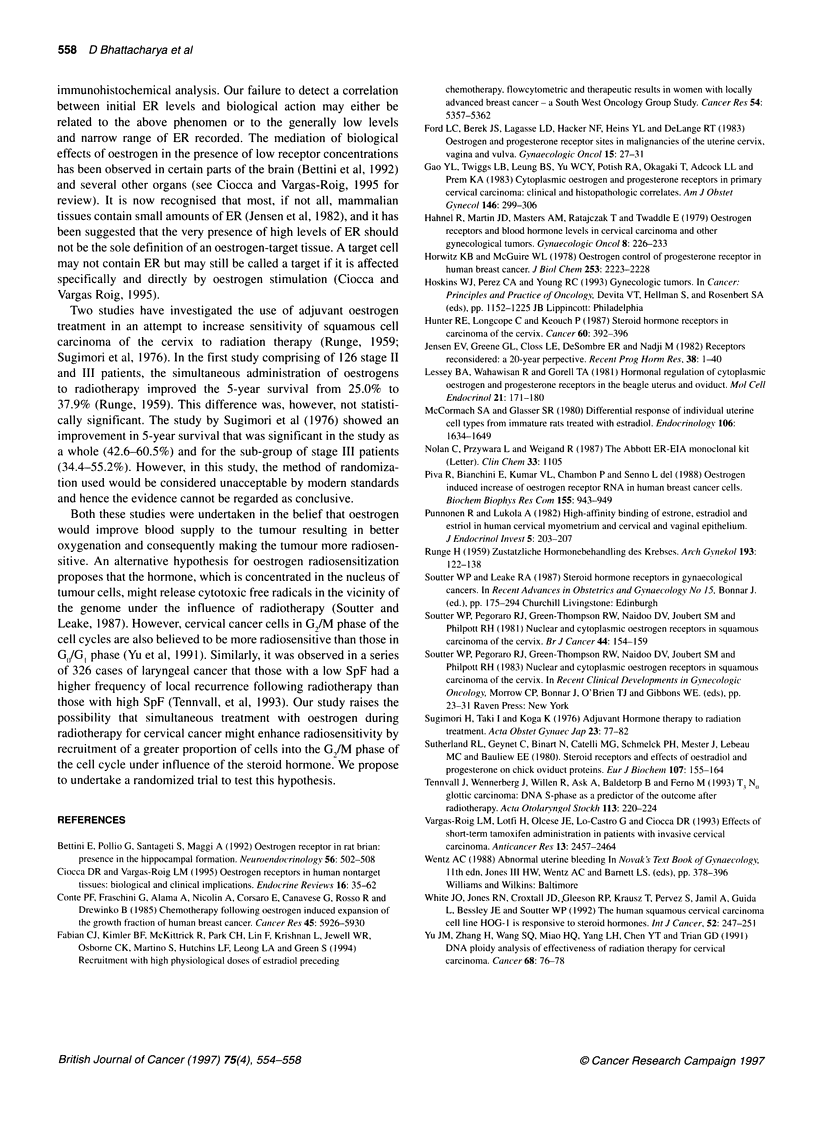# Oestrogen increases S-phase fraction and oestrogen and progesterone receptors in human cervical cancer in vivo.

**DOI:** 10.1038/bjc.1997.97

**Published:** 1997

**Authors:** D. Bhattacharya, A. Redkar, I. Mittra, U. Sutaria, K. D. MacRae

**Affiliations:** Department of Obstetrics and Gynecology, King Edward Memorial Hospital, Pune, India.

## Abstract

Although cancer of the cervix is traditionally considered not to be responsive to steroid hormones, an in vitro study has reported that the addition of oestrogen increased cellular proliferation in a cervix cancer cell line that was inhibited by progesterone. We investigated whether the reported in vitro effects of oestrogen and progesterone on cellular proliferation can be replicated in locally advanced cervical cancer in vivo and whether these effects, if any, are related to oestrogen and progesterone receptor (ER and PgR) content of the tumour. One hundred post-menopausal patients with locally advanced cervical cancer were systematically allocated by rotation to the four treatment groups: (1) control group receiving no treatment; (2) ethinyl oestradiol 50 micrograms: (3) norethisterone 5 mg: (4) a combination of ethinyl oestradiol and norethisterone. Hormone treatment (five doses) was given orally every 12 h. Tissue biopsies were taken before and 12 h after the last hormone treatment. S-phase fraction (SpF) was measured by flow cytometry, and ER and PgR were measured by enzyme immunoassay. Results were analysed using two-factor analysis of variance, the factors being oestrogen-absent or present- and progesterone-absent or present. The main effects of oestrogen were increases in SpF, ER and PgR, which were statistically significant (P = 0.0056, 0.0009 and 0.01 respectively), indicating that there is much greater change in these three parameters in the presence of oestrogen (mean changes 7.808%, 6.258 fmol mg-1 and 12.716 fmol mg-1 for SpF, ER and PgR respectively) than in its absence (mean change -1.986%,-3.041 fmol mg-1 and 1.736 fmol mg-1 respectively). The progestogen main effect and the oestrogen-progestogen interaction were not significant. The rise in SpF, ER and PgR in the presence of oestrogen had a correlation coefficient with the initial ER values of -0.0565, -0.2863 and -0.1230 respectively, none being statistically significant, suggesting that the oestrogen actions were not strictly related to baseline ER concentrations. The combined median baseline ER and PgR values of the four groups were 1.48 fmol mg-1 and 0.80 fmol mg-1 respectively. Our results show that oestrogen is capable of increasing SpF in locally advanced cervical cancer in vivo and may help to revive interest in the use of oestrogen as a radiosensitizing agent in the treatment of this disease.


British Journal of Cancer (1997) 75(4), 554–558
© 1997 Cancer Research Campaign

### Oestrogen increases S-phase fraction and oestrogen and progesterone receptors in human cervical cancer in vivo

**D Bhattacharya[1], A Redkar[2], I Mittra[2,3], U Sutaria[4] and KD MacRae[5]**

[1]Department of Obstetrics and Gynecology, King Edward Memorial Hospital, Pune 411 011, India; [2]Department of Laboratory Medicine and [3]Department of Surgery, Tata Memorial Hospital, Bombay 400 012, India; [4]Department of Obstetrics and Gynecology, Bairamjee Jeejeebhoy Medical College, Pune 411 001, India; [5]Department of Medical Statistics, Charing Cross and Westminster Medical School, London

**Summary** Although cancer of the cervix is traditionally considered not to be responsive to steroid hormones, an in vitro study has reported that the addition of oestrogen increased cellular proliferation in a cervix cancer cell line that was inhibited by progesterone. We investigated whether the reported in vitro effects of oestrogen and progesterone on cellular proliferation can be replicated in locally advanced cervical cancer in vivo and whether these effects, if any, are related to oestrogen and progesterone receptor (ER and PgR) content of the tumour. One hundred post-menopausal patients with locally advanced cervical cancer were systematically allocated by rotation to the four treatment groups: (1) control group receiving no treatment; (2) ethinyl oestradiol 50 μg; (3) norethisterone 5 mg; (4) a combination of ethinyl oestradiol and norethisterone. Hormone treatment (five doses) was given orally every 12 h. Tissue biopsies were taken before and 12 h after the last hormone treatment. S-phase fraction (SpF) was measured by flow cytometry, and ER and PgR were measured by enzyme immunoassay. Results were analysed using two-factor analysis of variance, the factors being oestrogen – absent or present – and progesterone – absent or present. The main effects of oestrogen were increases in SpF, ER and PgR, which were statistically significant ($P$= 0.0056, 0.0009 and 0.01 respectively), indicating that there is much greater change in these three parameters in the presence of oestrogen (mean changes 7.808 %, 6.258 fmol mg$^{-1}$ and 12.716 fmol mg$^{-1}$ for SpF, ER and PgR respectively) than in its absence (mean change –1.986 %, –3.041 fmol mg$^{-1}$ and 1.736 fmol mg$^{-1}$ respectively). The progestogen main effect and the oestrogen – progestogen interaction were not significant. The rise in SpF, ER and PgR in the presence of oestrogen had a correlation coefficient with the initial ER values of –0.0565, –0.2863 and –0.1230 respectively, none being statistically significant, suggesting that the oestrogen actions were not strictly related to baseline ER concentrations. The combined median baseline ER and PgR values of the four groups were 1.48 fmol mg$^{-1}$ and 0.80 fmol mg$^{-1}$ respectively. Our results show that oestrogen is capable of increasing SpF in locally advanced cervical cancer in vivo and may help to revive interest in the use of oestrogen as a radiosensitizing agent in the treatment of this disease.

**Keywords:** cervical cancer; S-phase fraction; oestrogen receptor; progesterone receptor; radiosensitisation

Carcinoma of the cervix is the commonest cancer in women in the developing world where most patients present at advanced stages when treatment with radiotherapy is not very effective (Hoskins et al, 1993). At least two studies have used adjuvant oestrogen treatment in an attempt to increase sensitivity of squamous cell carcinoma of the cervix to radiation therapy (Runge, 1959; Sugimori et al, 1976). One of these studies reported a significantly better survival in stage III patients in whom oestrogen was administered before and during radiation treatment than in the control group (Sugimori et al, 1976). Cancer of the cervix is conventionally not considered to be steroid hormone-responsive tissue. However, one report has claimed that a human cervical carcinoma cell line HOG-1 could be made to proliferate by the addition of oestradiol, which was inhibited by progesterone (White et al, 1992). In breast cancer, several studies of oestrogen priming have been conducted in an attempt to recruit cells in the proliferative phase of the cell cycle to enhance sensitivity of tumours to chemotherapy (Conte et al, 1985; Fabian et al, 1994). We conducted the present study to investigate whether short-term treatment with oral ethinyl oestradiol could increase the fraction of cells in the S-phase (SpF) in locally advanced cervical cancer in vivo. As progesterone has been shown to inhibit the effect of oestrogen on cell proliferation in a cervical carcinoma cell line (White et al, 1992), we also studied the effect on SpF of oral noresthisterone, a progestogen, alone or in combination with oestrogen. The effects of these hormones on oestrogen and progesterone receptors (ER and PgR) were also investigated.

*Received 7 May 1996*
*Revised 30 July 1996*
*Accepted 22 August 1996*

*Correspondence to:* I Mittra, Department of Laboratory Medicine and Surgery, Tata Memorial Hospital, Bombay - 400 012, India

### MATERIALS AND METHODS

Between January 1993 and August 1994, 100 post-menopausal women with locally advanced carcinoma of the cervix (FIGO stage IIB or above) were recruited into the study. Of these, 49% were FIGO stage IIB, 47% were stage IIIB and 4% were stage IVA. The ages of these patients varied between 45 and 72 years, with a mean of 55.34 ± 7.55 (s.d.) years. Histopathological biopsy reports revealed that 93% were squamous cell carcinomas, 6% were adenosquamous carcinomas and 1% adenocarcinoma. Nine per cent of the carcinomas were of histological grade I, 62% of grade II and 29% of grade III.

**Figure 1** Scatter plots showing changes in SpF, ER and PgR in response to hormones. (**A**) Control group; (**B**) oestrogen-treated group; (**C**) progestogen-treated group; (**D**) combination group. For dose see text

© Cancer Research Campaign 1997

**Table 1** Table of means

| | No E (*n*) | E (*n*) | No E + E (*n*) |
|---|---|---|---|
| SpF difference (%) | | | |
| No P | 0.231 (25) | 8.871 (24) | 4.55[a] (49) |
| P | –4.204 (23) | 6.745 (20) | 1.270[a] (43) |
| No P + P | –1.986[b] (48) | 7.808[b] (44) | |
| ER difference (fmol mg$^{-1}$) | | | |
| No P | –3.800 | 7.677 | 1.938[a] |
| P | –2.281 | 4.839 | 1.279[a] |
| No P + P | –3.041[b] | 6.258[b] | |
| PgR difference (fmol mg$^{-1}$) | | | |
| No P | –0.346 | 17.277 | 8.465[a] |
| P | 3.818 | 8.154 | 5.986[a] |
| No P + P | 1.736[b] | 12.716[b] | |

[a]Comparison of effects of progestogen, ignoring oestrogen. [b]Comparison of effects of oestrogen, ignoring progestogen. E, Oestrogen; P, progestogen.

**Table 2** Summary analyses of variance

| | Sum of squares | d.f. | Mean square | *e* | *P*-value |
|---|---|---|---|---|---|
| SpF difference | | | | | |
| E | 2190.83 | 1 | 2190.83 | 8.07 | 0.0056 |
| P | 245.84 | 1 | 245.84 | 0.91 | 0.3439 |
| EP | 30.47 | 1 | 30.47 | 0.11 | 0.7384 |
| Error | 23894.34 | 88 | 271.53 | | |
| ER difference | | | | | |
| E | 1974.81 | 1 | 1974.81 | 11.93 | 0.0009 |
| P | 9.93 | 1 | 9.93 | 0.06 | 0.81 |
| EP | 108.40 | 1 | 108.40 | 0.66 | 0.42 |
| Error | 14562.62 | 88 | 165.48 | | |
| PgR difference | | | | | |
| E | 2753.19 | 1 | 2753.19 | 6.86 | 0.01 |
| P | 140.37 | 1 | 140.37 | 0.35 | 0.56 |
| EP | 1008.10 | 1 | 1008.10 | 2.51 | 0.12 |
| Error | 35315.76 | 88 | 401.32 | | |

E, Oestrogen; P, progestogen; EP, interaction of E and P.

After general examination for fitness, all patients in the study were admitted to hospital to ensure compliance to the treatment regimens. After exposing the cervical growth with the help of a Cusco speculum, a punch biopsy was taken from the periphery of the tumour avoiding the central necrotic area. A part of the primary biopsy (Bx 1) was sent for histopathology and the remainder kept frozen at –80°C in 12% dimethyl sulphoxide in Dulbecco's modified Eagle medium for flow-cytometric (FCM) analysis. Another part of the specimen was wrapped in aluminium foil and stored at –80°C for ER and PgR assays.

The patients were not randomized but systematically allocated to the following four treatment arms strictly by rotation, i.e. (1) no treatment, (2) ethinyl oestradiol 50 μg orally every 12 h × five doses, (3) norethisterone 5 mg orally every 12 h × five doses, (4) combination of ethinyl oestradiol and norethisterone orally every 12 h × five doses. The above dose levels were chosen as these are within the therapeutic range for these hormones when used for conditions such as dysfunctional uterine bleeding (Wentz, 1988). Twelve hours after the last hormone dose, a second biopsy (Bx 2) was taken and stored in the same fashion for flow cytometry analysis and ER and PgR assays. Radiation therapy was commenced immediately after the second biopsy.

This study was conducted after obtaining approval from the institutional review body, and a written informed consent was obtained from all participating women.

Flow cytometry was performed using the Epics Profile machine (Coulter, Hialea, FL USA). A single-cell suspension was prepared from each frozen tumour sample, fixed in 70% chilled ethanol and stained with propidium iodide (50 μg ml$^{-1}$). Approximately 1–2 × $10^6$ cells were used for each analysis. Normal human peripheral blood lymphocytes separated on Ficoll were used as a diploid reference standard in each assay batch. The data were stored in a histogram mode on hard disk for later retrieval. Cell cycle analysis was performed using the multicycle software from Phoenix Flow Systems, San Diego, CA, USA. For every case both ploidy status and SpF were measured. Eight biopsy samples (either Bx 1 or Bx 2) generated unsatisfactory histograms that could not be interpreted. These eight cases were excluded from the study.

ER and PgR assay was performed by an enzyme immunoassay (EIA) technique using the Abbot kit (Nolan et al, 1987). The method involves the use of two distinct antibodies to each receptor in a 'sandwich' technique. Bx 1 and Bx 2 samples from each patient were always analysed in the same assay batch.

Of the 100 patients, 92 samples were evaluable for SpF, ER and PgR for both Bx 1 and Bx 2. Consequently, there were 25 patients in the control group, 24 in the oestrogen-treated group, 23 in the progestogen-treated group and 20 patients in the combined treatment group.

### RESULTS

Figure 1 depicts scatter plots of SpF, ER and PgR values of Bx 1 and Bx 2 in the four treatment groups. The changes in SpF, ER and PgR in the four groups were analysed statistically using two-factor analyses of variance, the factors being oestrogen (E) – absent or present – and progestogen (P) – absent or present. These give tests of the main effects of E and P and a test of the interaction of E and P (EP). The calculations were carried out on the raw untransformed data and were checked using logarithmically transformed data because of the presence of positive skew in the raw data. The results of the logarithmically transformed data analyses will not be shown separately, as the conclusion on these analyses were identical to those from the raw data analyses.

The main effects of oestrogen and progestogen on SpF, ER and PgR are shown in Table 1. Table 2 summarizes the analyses of variance of differences in SpF, ER and PgR.

### S-phase fraction

A comparison of the means of no E vs E groups gives the main effect of oestrogen, that is, the mean changes in SpF without and with oestrogen ignoring progestogen (Table 1). The mean per cent SpF difference without oestrogen (combining the no progestogen and progestogen groups) is 1.986, while with oestrogen the mean change is 7.808. Similarly, the main effect of progestogen on SpF is seen from the comparison of mean per cent changes without and with progestogen, ignoring oestrogen. The overall mean SpF difference without progestogen is 4.55 and with progestogen is 1.270.

Table 2 gives the summary analysis of variance of the data. It shows that the main effect of E on SpF is statistically significant ($P$ = 0.0056), indicating that there is a much greater change in SpF in the presence of oestrogen (mean change 7.808%) than in its absence (mean change –1.986%, Table 1). The P main effect and

© Cancer Research Campaign 1997

the EP interaction are both non-significant ($P = 0.3439$ and 0.7384 respectively); the former being consistent with there being no progestogen effect and the latter with there being no modification by progestogen of the oestrogen effect.

The rise in SpF in the presence of oestrogen has a correlation coefficient of –0.057 with the initial ER value and 0.227 with the initial PgR value, neither being statistically significant. Similarly, correlation coefficient of the SpF rise in the presence of oestrogen with post-treatment (Bx 2) values of ER and PgR were also statistically non-significant (–0.2265 and 0.0599 respectively). The combined median baseline ER and PgR values of the four groups were 1.48 fmol $mg^{-1}$ and 0.80 fmol $mg^{-1}$ respectively. Only 17.40% of ER and 4.35% of PgR were above the conventional cut-off level of 10 fmol $mg^{-1}$ which is used in breast cancer to distinguish receptor-positive and receptor-negative tumours.

### Oestrogen receptor

The main effect of E on ER is statistically significant ($P = 0.0009$, Table 2), indicating that there is a much greater change in ER in the presence of oestrogen (mean change 6.258 fmol $mg^{1}$) than in its absence (mean change 3.041 fmol $mg^{1}$, Table 1). The P main effect on ER and the EP interaction are both non-significant ($P =$ 0.81 and 0.42 respectively); the former being consistent with there being no progestogen effect and the latter with there being no modification by progestogen of the oestrogen effect.

The rise in ER in the presence of oestrogen has a correlation coefficient of –0.2863 with initial ER value and –0.1490 with initial PgR value, neither being statistically significant.

### Progesterone receptor

The main effect of E on PgR is statistically significant ($P = 0.01$, Table 2), indicating that there is a much greater change in PgR in the presence of oestrogen (mean change 12.716 fmol $mg^{1}$) than in its absence (mean change 1.736 fmol $mg^{1}$, Table 1). The P main effect on PgR and the EP interaction are both non-significant ($P =$ 0.56 and 0.12 respectively); the former being consistent with there being no progestogen effect and the latter with there being no modification by progestogen of the oestrogen effect.

The rise in PgR in the presence of oestrogen has a correlation coefficient of –0.1230 with initial ER and –0.0254 with initial PgR value, neither being statistically significant.

### DISCUSSION

Our study demonstrates that short-term treatment with ethinyl oestradiol given orally in 'physiological' doses causes a significant rise in SpF, ER and PgR concentrations in post-menopausal women with locally advanced cervical carcinoma. Norethisterone, a progestogen, had no effect on the above biological parameters, nor did it have any interaction with the oestrogen effect when given in combination with ethinyl oestradiol. To our knowledge, a proliferative effect of oestradiol on cervical cancer in vivo has, so far, not been demonstrated. The lack of a progestogen effect in our study might have been as a result of the dose level that was used. It remains to be seen whether progestogen in higher doses can suppress SpF or can counteract the proliferative effect of oestrogen.

We observed that oestrogen administration caused a significant rise in PgR levels in cervical cancer. Although it is well established that oestrogen induces PgR in human breast cancer (Horwitz and McGuire, 1978), such an effect had so far not been demonstrated in cervical cancer. We also observed that oestrogen administration caused the induction of its own receptor. This phenomenon has not been widely recognized in humans, although oestrogen-stimulated induction of its own receptor has been reported in several animal models (McCormach and Glasser, 1980; Sutherland et al, 1980; Lessey et al, 1981). Piva et al (1988) have demonstrated an increase in ER mRNA in human breast cancer cell lines cultured with oestradiol. ER was shown to increase in normal cervicovaginal epithelium in four post-menopausal women after treatment with vaginal oestrogen pesseries (Punnonen and Lukola, 1982). The highly significant increase in ER in patients receiving oestrogen was, however, unaffected by the simultaneous administration of progestogen in the dose levels used.

Although cancer of the cervix is considered not to be responsive to steroid hormones, the normal cervix is known to respond actively to sex steroids (Soutter and Leake, 1987). The presence of oestrogen receptor was reported in all the samples of normal cervical tissue examined in premenopausal woman (Soutter et al, 1981, 1983). Several authors have measured ER and PgR levels in cervical cancer (see Soutter and Leake, 1987 for review). However, the proportion showing their presence have been variable. For example, Vargas et al (1993) and Hahnel et al (1979) reported low or undetectable levels of ER and PgR in cervical cancer using immunohistochemical and ligand-binding assays respectively. Other workers using the latter technique have reported somewhat higher levels (Ford et al, 1983; Gao et al, 1983; Hunter et al, 1987). The differences between studies may be the result of differences in method for tissue collection, differences in storage conditions, differences in assay techniques and perhaps differences in patient populations. Soutter and Leake (1987) have reported better preservation of steroid receptors when tissues are stored in a hyperosmolar glycerol buffer rather than in liquid nitrogen. In their study, using these storage conditions, oestrogen receptors were found in 45.2% of 73 squamous tumours. In our study, in which an EIA technique was used, ER and PgR were found in 90.2% and 81.5% of tumours; but the values were generally low with median figures of 1.48 fmol $mg^{-1}$ and 0.80 fmol $mg^{-1}$ respectively. We scored all tissues in which ER and PgR values could be recorded as evidence for presence of receptors. Perhaps a better alternative would have been to include squamous cell carcinomas from non-reproductive tissues as negative controls to determine a cut-off value. It has been generally observed that ER and PgR levels are higher in adenocarcinomas than in squamous cell carcinomas of the cervix (Hahnel et al, 1979; Ford et al, 1983). The relatively low receptor values observed in our study may be related to the fact that 93% of the tumours included in our study were of squamous cell origin. We did not find any relationship between steroid receptor levels and stage of disease.

Although the levels of ER and PgR observed in cervix cancer are relatively low compared with those detected in breast cancer, these levels were nevertheless apparently sufficient to bring about the biological changes observed. Our finding that oestrogen administration caused cellular proliferation and the induction of PgR clearly indicates that the action of oestradiol was mediated via the ER pathway. It is possible that, in spite of low levels of ER, there may be very important differences in ER contents among cells in the tissue biopsy, and that those cells that express ER are the cells that respond to increases in SpF following oestrogen treatment. This issue would need to be resolved by simultaneous analysis of ER and cells that are proliferating, e.g. by double

© Cancer Research Campaign 1997

immunohistochemical analysis. Our failure to detect a correlation between initial ER levels and biological action may either be related to the above phenomen or to the generally low levels and narrow range of ER recorded. The mediation of biological effects of oestrogen in the presence of low receptor concentrations has been observed in certain parts of the brain (Bettini et al, 1992) and several other organs (see Ciocca and Vargas-Roig, 1995 for review). It is now recognised that most, if not all, mammalian tissues contain small amounts of ER (Jensen et al, 1982), and it has been suggested that the very presence of high levels of ER should not be the sole definition of an oestrogen-target tissue. A target cell may not contain ER but may still be called a target if it is affected specifically and directly by oestrogen stimulation (Ciocca and Vargas Roig, 1995).

Two studies have investigated the use of adjuvant oestrogen treatment in an attempt to increase sensitivity of squamous cell carcinoma of the cervix to radiation therapy (Runge, 1959; Sugimori et al, 1976). In the first study comprising of 126 stage II and III patients, the simultaneous administration of oestrogens to radiotherapy improved the 5-year survival from 25.0% to 37.9% (Runge, 1959). This difference was, however, not statistically significant. The study by Sugimori et al (1976) showed an improvement in 5-year survival that was significant in the study as a whole (42.6–60.5%) and for the sub-group of stage III patients (34.4–55.2%). However, in this study, the method of randomization used would be considered unacceptable by modern standards and hence the evidence cannot be regarded as conclusive.

Both these studies were undertaken in the belief that oestrogen would improve blood supply to the tumour resulting in better oxygenation and consequently making the tumour more radiosensitive. An alternative hypothesis for oestrogen radiosensitization proposes that the hormone, which is concentrated in the nucleus of tumour cells, might release cytotoxic free radicals in the vicinity of the genome under the influence of radiotherapy (Soutter and Leake, 1987). However, cervical cancer cells in $G_2$/M phase of the cell cycles are also believed to be more radiosensitive than those in $G_0/G_1$ phase (Yu et al, 1991). Similarly, it was observed in a series of 326 cases of laryngeal cancer that those with a low SpF had a higher frequency of local recurrence following radiotherapy than those with high SpF (Tennvall, et al, 1993). Our study raises the possibility that simultaneous treatment with oestrogen during radiotherapy for cervical cancer might enhance radiosensitivity by recruitment of a greater proportion of cells into the $G_2$/M phase of the cell cycle under influence of the steroid hormone. We propose to undertake a randomized trial to test this hypothesis.

### REFERENCES

Bettini E, Pollio G, Santageti S, Maggi A (1992) Oestrogen receptor in rat brian: presence in the hippocampal formation. *Neuroendocrinology* **56**: 502–508

Ciocca DR and Vargas-Roig LM (1995) Oestrogen receptors in human nontarget tissues: biological and clinical implications. *Endocrine Reviews* **16**: 35–62

Conte PF, Fraschini G, Alama A, Nicolin A, Corsaro E, Canavese G, Rosso R and Drewinko B (1985) Chemotherapy following oestrogen induced expansion of the growth fraction of human breast cancer. *Cancer Res* **45**: 5926–5930

Fabian CJ, Kimler BF, McKittrick R, Park CH, Lin F, Krishnan L, Jewell WR, Osborne CK, Martino S, Hutchins LF, Leong LA and Green S (1994) Recruitment with high physiological doses of estradiol preceding chemotherapy, flowcytometric and therapeutic results in women with locally advanced breast cancer – a South West Oncology Group Study. *Cancer Res* **54**: 5357–5362

Ford LC, Berek JS, Lagasse LD, Hacker NF, Heins YL and DeLange RT (1983) Oestrogen and progesterone receptor sites in malignancies of the uterine cervix, vagina and vulva. *Gynaecologic Oncol* **15**: 27–31

Gao YL, Twiggs LB, Leung BS, Yu WCY, Potish RA, Okagaki T, Adcock LL and Prem KA (1983) Cytoplasmic oestrogen and progesterone receptors in primary cervical carcinoma: clinical and histopathologic correlates. *Am J Obstet Gynecol* **146**: 299–306

Hahnel R, Martin JD, Masters AM, Ratajczak T and Twaddle E (1979) Oestrogen receptors and blood hormone levels in cervical carcinoma and other gynecological tumors. *Gynaecologic Oncol* **8**: 226–233

Horwitz KB and McGuire WL (1978) Oestrogen control of progesterone receptor in human breast cancer. *J Biol Chem* **253**: 2223–2228

Hoskins WJ, Perez CA and Young RC (1993) Gynecologic tumors. In *Cancer: Principles and Practice of Oncology*, Devita VT, Hellman S, and Rosenbert SA (eds), pp. 1152–1225 JB Lippincott: Philadelphia

Hunter RE, Longcope C and Keouch P (1987) Steroid hormone receptors in carcinoma of the cervix. *Cancer* **60**: 392–396

Jensen EV, Greene GL, Closs LE, DeSombre ER and Nadji M (1982) Receptors reconsidered: a 20-year perpective. *Recent Prog Horm Res*, **38**: 1–40

Lessey BA, Wahawisan R and Gorell TA (1981) Hormonal regulation of cytoplasmic oestrogen and progesterone receptors in the beagle uterus and oviduct. *Mol Cell Endocrinol* **21**: 171–180

McCormach SA and Glasser SR (1980) Differential response of individual uterine cell types from immature rats treated with estradiol. *Endocrinology* **106**: 1634–1649

Nolan C, Przywara L and Weigand R (1987) The Abbott ER-EIA monoclonal kit (Letter). *Clin Chem* **33**: 1105

Piva R, Bianchini E, Kumar VL, Chambon P and Senno L del (1988) Oestrogen induced increase of oestrogen receptor RNA in human breast cancer cells. *Biochem Biophys Res Com* **155**: 943–949

Punnonen R and Lukola A (1982) High-affinity binding of estrone, estradiol and estriol in human cervical myometrium and cervical and vaginal epithelium. *J Endocrinol Invest* **5**: 203–207

Runge H (1959) Zustatzliche Hormonebehandling des Krebses. *Arch Gynekol* **193**: 122–138

Soutter WP and Leake RA (1987) Steroid hormone receptors in gynaecological cancers. In *Recent Advances in Obstetrics and Gynaecology No 15*, Bonnar J. (ed.), pp. 175–294 Churchill Livingstone: Edinburgh

Soutter WP, Pegoraro RJ, Green-Thompson RW, Naidoo DV, Joubert SM and Philpott RH (1981) Nuclear and cytoplasmic oestrogen receptors in squamous carcinoma of the cervix. *Br J Cancer* **44**: 154–159

Soutter WP, Pegoraro RJ, Green-Thompson RW, Naidoo DV, Joubert SM and Philpott RH (1983) Nuclear and cytoplasmic oestrogen receptors in squamous carcinoma of the cervix. In *Recent Clinical Developments in Gynecologic Oncology*, Morrow CP, Bonnar J, O'Brien TJ and Gibbons WE. (eds), pp. 23–31 Raven Press: New York

Sugimori H, Taki I and Koga K (1976) Adjuvant Hormone therapy to radiation treatment. *Acta Obstet Gynaec Jap* **23**: 77–82

Sutherland RL, Geynet C, Binart N, Catelli MG, Schmelck PH, Mester J, Lebeau MC and Bauliew EE (1980). Steroid receptors and effects of oestradiol and progesterone on chick oviduct proteins. *Eur J Biochem* **107**: 155–164

Tennvall J, Wennerberg J, Willen R, Ask A, Baldetorp B and Ferno M (1993) $T_3$ $N_0$ glottic carcinoma: DNA S-phase as a predictor of the outcome after radiotherapy. *Acta Otolaryngol Stockh* **113**: 220–224

Vargas-Roig LM, Lotfi H, Olcese JE, Lo-Castro G and Ciocca DR (1993) Effects of short-term tamoxifen administration in patients with invasive cervical carcinoma. *Anticancer Res* **13**: 2457–2464

Wentz AC (1988) Abnormal uterine bleeding In *Novak's Text Book of Gynaecology*, 11th edn, Jones III HW, Wentz AC and Barnett LS. (eds), pp. 378–396 Williams and Wilkins: Baltimore

White JO, Jones RN, Croxtall JD, Gleeson RP, Krausz T, Pervez S, Jamil A, Guida L, Bessley JE and Soutter WP (1992) The human squamous cervical carcinoma cell line HOG-1 is responsive to steroid hormones. *Int J Cancer*, **52**: 247–251

Yu JM, Zhang H, Wang SQ, Miao HQ, Yang LH, Chen YT and Trian GD (1991) DNA ploidy analysis of effectiveness of radiation therapy for cervical carcinoma. *Cancer* **68**: 76–78

 © Cancer Research Campaign 1997